# Consequences of Gestational Diabetes in an Urban Hospital in Viet Nam: A Prospective Cohort Study

**DOI:** 10.1371/journal.pmed.1001272

**Published:** 2012-07-24

**Authors:** Jane E. Hirst, Thach S. Tran, My An T. Do, Jonathan M. Morris, Heather E. Jeffery

**Affiliations:** 1 Kolling Institute of Medical Research, University of Sydney, Sydney, Australia; 2 Department of Obstetrics & Gynaecology, Royal North Shore Hospital, St Leonards, Australia; 3 Hung Vuong Hospital, Ho Chi Minh City, Viet Nam; 4 Australian Research Centre for Health of Women and Babies, University of Adelaide, Adelaide, Australia; 5 International Women and Children's Health, Sydney School of Public Health, University of Sydney, Sydney, Australia; 6 Department of Neonatology, Royal Prince Alfred Hospital, Sydney, Australia; Cambridge University, United Kingdom

## Abstract

**Background:**

Gestational diabetes mellitus (GDM) is increasing and is a risk for type 2 diabetes. Evidence supporting screening comes mostly from high-income countries. We aimed to determine prevalence and outcomes in urban Viet Nam. We compared the proposed International Association of the Diabetes and Pregnancy Study Groups (IADPSG) criterion, requiring one positive value on the 75-g glucose tolerance test, to the 2010 American Diabetes Association (ADA) criterion, requiring two positive values.

**Methods and Findings:**

We conducted a prospective cohort study in Ho Chi Minh City, Viet Nam. Study participants were 2,772 women undergoing routine prenatal care who underwent a 75-g glucose tolerance test and interview around 28 (range 24–32) wk. GDM diagnosed by the ADA criterion was treated by local protocol. Women with GDM by the IADPSG criterion but not the ADA criterion were termed “borderline” and received standard care. 2,702 women (97.5% of cohort) were followed until discharge after delivery. GDM was diagnosed in 164 participants (6.1%) by the ADA criterion, 550 (20.3%) by the IADPSG criterion. Mean body mass index was 20.45 kg/m^2^ in women with out GDM, 21.10 in women with borderline GDM, and 21.81 in women with GDM, *p*<0.001. Women with GDM and borderline GDM were more likely to deliver preterm, with adjusted odds ratios (aORs) of 1.49 (95% CI 1.16–1.91) and 1.52 (1.03–2.24), respectively. They were more likely to have clinical neonatal hypoglycaemia, aORs of 4.94 (3.41–7.14) and 3.34 (1.41–7.89), respectively. For large for gestational age, the aORs were 1.16 (0.93–1.45) and 1.31 (0.96–1.79), respectively. There was no significant difference in large for gestational age, death, severe birth trauma, or maternal morbidity between the groups. Women with GDM underwent more labour inductions, aOR 1.51 (1.08–2.11).

**Conclusions:**

Choice of criterion greatly affects GDM prevalence in Viet Nam. Women with GDM by the IADPSG criterion were at risk of preterm delivery and neonatal hypoglycaemia, although this criterion resulted in 20% of pregnant women being positive for GDM. The ability to cope with such a large number of cases and prevent associated adverse outcomes needs to be demonstrated before recommending widespread screening.

*Please see later in the article for the Editors' Summary.*

## Abstract 2

Jane Hirst and colleagues determined the prevalence and outcome of gestational diabetes mellitus in urban Vietnam and found that choice of criteria greatly affected prevalence, and has implications for the ability of the health system to cope with the number of cases.

## Editors' Summary

**Background:**

Gestational diabetes mellitus (GDM) is diabetes that is first diagnosed during pregnancy. Like other types of diabetes, it is characterized by high levels of sugar (glucose) in the blood. Blood-sugar levels are usually controlled by insulin, which is made by the pancreas. Hormonal changes during pregnancy and the baby's growth demands increase a pregnant woman's insulin needs, and if her pancreas cannot make enough insulin, GDM develops, usually in mid-pregnancy. Risk factors for GDM include a high body mass index (a measure of body fat), excessive weight gain or lack of physical activity during pregnancy, and glucose intolerance (an indicator of diabetes that is measured using the “oral glucose tolerance test”). GDM increases the risk of premature delivery, induced delivery, and having a large-for-gestational-age baby (gestation is the time during which the baby develops within the mother). It also increases the baby's risk of having low blood sugar (neonatal hypoglycemia). GDM, which can often be controlled by exercise and diet, usually disappears after pregnancy but increases the risk of diabetes developing later in both mother and baby.

**Why Was This Study Done?:**

The prevalence (occurrence) of diabetes is increasing rapidly, particularly in low/middleincome countries as they become more affluent. Because GDM increases the subsequent risk of diabetes, some experts believe that screening for GDM should be included in prenatal care as part of diabetes preventative strategies. However, most of the evidence supporting GDM screening comes from high-income countries, so in this prospective cohort study (a study that analyses associations between the baseline characteristics of a group of patients and outcomes), the researchers investigate the prevalence of GDM (diagnosed using the oral glucose tolerance test) and the consequences of GDM among women attending an urban hospital in Viet Nam, a low/middle-income country. An oral glucose tolerance test measures a patient's blood-sugar level after an overnight fast, and one and two hours after consuming a sugary drink. The International Association of the Diabetes and Pregnancy Study Groups (IADPSG) and the American Diabetes Association (ADA) guidelines state, respectively, that one and two of these blood-sugar measurements must be abnormally high for a diagnosis of GDM. In this study, the researchers use both guidelines to diagnose GDM.

**What Did the Researchers Do and Find?:**

Nearly 3,000 women who attended the hospital for routine prenatal care had a glucose tolerance test at around 28 weeks' gestation and were followed until discharge after delivery. Women who had GDM diagnosed by the ADA criterion were referred for dietary advice and glucose monitoring. Those diagnosed by the IADPSG criterion only were described as having “borderline” GDM and received standard prenatal care. GDM was diagnosed in 6.1% and 20.3% of the women using the ADA and IADPSG criteria, respectively. After allowing for other factors that might have affected outcomes, compared to women without GDM, women with GDM or borderline GDM were more likely to deliver prematurely, and their babies were more likely to have neonatal hypoglycemia. Also, women with GDM (but not borderline GDM) were more likely to have their labor induced than women without GDM.

**What Do These Findings Mean?:**

These findings show that the criterion used to diagnose GDM markedly affected the prevalence of GDM among pregnant women attending this Vietnamese hospital—the use of the IADPSG criterion more than tripled the prevalence of GDM and meant that a fifth of the study participants were diagnosed as having GDM. Importantly, the findings also show that GDM diagnosed using the IADPSG criterion was associated with an increased risk of preterm delivery and neonatal hypoglycemia. Although these findings may not be generalizable to other settings within Viet Nam or to other countries, they highlight the need to demonstrate that sufficient resources are available to cope with an increased GDM burden before recommending widespread screening using the IADPSG criterion. Moreover, because the long-term significance of GDM diagnosed using the IADPSG criterion is not known, all the potential benefits and harms and the costs of screening and treating GDM in low-income settings need to be further investigated before any recommendation for “universal” GDM screening is made.

**Additional Information:**

Please access these websites via the online version of this summary at http://dx.doi.org/10. 1371/journal.pmed.1001272.

- The US National Institute of Diabetes and Digestive and Kidney Diseases provides information for patients on diabetes and on gestational diabetes (in English and Spanish)
- The UK National Health Service Choices website also provides information for patients about diabetes and about gestational diabetes, including links to other useful resources
- The American Diabetes Association also provides detailed information for patients and professionals about all aspects of diabetes, including gestational diabetes (in English and Spanish)
- The International Association of the Diabetes and Pregnancy Study Groups (IADPSG) 2010 recommendations on the diagnosis and classification of gestational diabetes are available
- The charity Diabetes UK provides detailed information for patients and carers, including information on gestational diabetes; its blog includes a personal story about gestational diabetes, and its website includes a selection of other stories from people with diabetes; the charity Healthtalkonline also has an interview describing a personal experience of gestational diabetes
- MedlinePlus provides links to additional resources on diabetes and on gestational diabetes (in English and Spanish)

## Introduction

Diabetes is rising globally. It is predicted that 438 million people will be living with diabetes by 2020, and 50% of these will reside in Asia [1]. Countries undergoing social, economic, and nutritional transition are experiencing the greatest increase in prevalence, with commensurate impact on health service delivery. Gestational diabetes mellitus (GDM) is associated with a 7-fold increased risk of developing type 2 diabetes in the future [2], thus identification could have importance for preventative health strategies. The adverse maternal and foetal effects of GDM are well known [3]. The Hyperglycaemia and Adverse Pregnancy Outcomes (HAPO) cohort study involving over 23,000 women demonstrated strong evidence of a continuous rather than threshold relationship with rising glycaemia [4]. Such evidence, in addition to the findings of two randomised trials supporting treatment for mild hyperglycaemia [3],[5], has prompted the International Association of the Diabetes and Pregnancy Study Groups (IADPSG) to revise screening and diagnostic criteria for GDM. It has been proposed that all pregnant women undergo a 75-g oral glucose tolerance test (OGTT) around 28 wk gestation, with the threshold for diagnosis of GDM based on increased perinatal risk rather than future risk of developing diabetes or non-pregnancy values [6]. There is concern that these new guidelines will increase the number of women diagnosed with GDM, with a possible increase in iatrogenic intervention, with more pregnancies labelled “high risk” [7]. It is pertinent that evidence for the effectiveness of screening and treatment of GDM comes from high-income settings [8],[9], and direct transfer into other contexts may not replicate the same benefits.

Viet Nam is a low/middle-income Southeast Asian country with a population of approximately 87 million. In 2009, gross domestic product per capita was estimated at US$1,191 [10]. Type 2 diabetes is rising. In 2010 a population-based study found a prevalence of 11% in adult women residing in Ho Chi Minh City [11]. Few data are available on the prevalence of GDM in Viet Nam and associated perinatal outcomes, although rates in expatriate Vietnamese women are known to be high [12]. In southern Viet Nam the rate of institutional delivery and antenatal care is high [13], yet screening for GDM is not uniform and, if it occurs, is risk factor based. State-run maternity hospitals are frequently overcrowded and lack support from nutritionists and diabetes educators.

The aim of this study was to determine prevalence of GDM and follow women through pregnancy to assess the perinatal outcomes associated with gestational diabetes in urban Viet Nam. Given current international controversy over the optimal diagnostic criteria to use in order to prevent complications from GDM, we compared outcomes in women and their babies diagnosed with GDM by the American Diabetes Association (ADA) 2010 criterion [14] to outcomes for the additional women and babies that would be diagnosed by the less stringent IADPSG criterion [6].

## Methods

### Ethics Statement

Ethical approval was obtained from the University of Sydney (Human Research Ethics Committee approval number 13200) and the Hung Vuong Hospital Ethics Approval Board (approval number 725/Q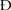-BVHV) prior to commencement of the study. All study participants were given written and oral information about the study and provided written informed consent to participate and have birth outcomes reviewed after delivery. There was no financial or other incentive to join the study, however, as Viet Nam has a user-pays health system; the cost of the OGTT was covered by the study protocol.

### Study Setting and Population

We carried out a prospective cohort study assessing the perinatal outcomes associated with GDM in Hung Vuong Hospital, Ho Chi Minh City. This hospital serves as a local and referral hospital for women in the city and surrounding provinces and conducted around 35,000 deliveries in 2010. Around a quarter of the women who deliver in the hospital are local women receiving routine antenatal care through the outpatient departments, and these women represented the target population for this study. Women referred from other hospitals or private clinics for management of antenatal complications or delivery were excluded, as it was felt they would not reflect population norms.

Women were approached in the antenatal outpatient department by one of three trained research midwives and screened for eligibility. Women were eligible if they were receiving antenatal care through the hospital outpatient departments, were aged over 18 y, had confirmed gestation between 24 and 32 wk (by early ultrasound or certain last menstrual period date), had a singleton pregnancy, planned to deliver in the hospital, and were not known to have diabetes.

Participants were recruited from 1 December 2010 to 31 March 2011, and all women had delivered by 21 August 2011. All study participants underwent a 75-g, 2-h OGTT between 24 and 32 wk gestation, with testing as close to 28 wk as possible. Women were given instructions to fast from midnight and present in the morning for testing. Blood samples were collected fasting, 1 h, and 2 h after ingestion of 75 g of anhydrous glucose dissolved in 200 ml of water.

We aimed to follow through all women screened around 28 wk of pregnancy until discharge from hospital following delivery (usually within 5 d of birth). Women with GDM were approached to undergo a short interview post-delivery to determine the method of monitoring of glycaemia and management of GDM.

### Data Collection

To assess sociodemographic characteristics and medical risk factors for GDM, women completed a structured, 10-min interview at the time of oral glucose tolerance testing conducted by one of three trained research midwives. Weight, height, and blood pressure were determined from the first antenatal visit record and measured again at the time of the OGTT. This interview solicited information about known and possible risk factors for GDM as well as basic pregnancy, health, social, and demographic information. The interview was trialled on around 100 women for acceptability and applicability prior to commencement of the main study.

To assess GDM status, blood samples were collected from the ante cubital fossa and processed within 1 h of collection using the glucose hexokinase enzymatic method (Roche/cobase c system c501). Calibration was performed with each new batch of reagent or every 2 d, whichever was sooner, as per the manufacturer's instructions. If values were obtained outside the reference range, recalibration was performed and the samples retested to confirm the result.

To assess perinatal outcomes, all medical records of mothers and their babies were reviewed after hospital discharge, and data were extracted by research midwives using a standardised form. Phone calls were made to women lost to follow-up to obtain basic birth outcome information.

The ADA criterion for GDM [14] requires two or more of the following glucose values: fasting glucose ≥95 mg/dl (5.3 mmol/l), 1-h glucose ≥180 (10.0), and/or 2-h glucose ≥155 (8.6). The IADPSG criterion [6] requires only one of the following glucose values: fasting glucose ≥92 mg/dl (5.1 mmol/l), 1-h glucose ≥180 (10.0), or 2-h glucose ≥153 (8.5). Three strata were defined: women with GDM according to both the ADA criterion and the IADPSG criterion (considered as having GDM), women with GDM by the IADPSG criterion alone (considered “borderline”), and women without GDM by either criterion (considered “normal”).

Only women with GDM according to the ADA criterion were notified of their diagnosis and referred for dietary advice and glucose monitoring. All other women received standard antenatal care. The hospital had a loan system for home blood glucose monitors, although women pay a deposit for disposables and many prefer to come to the hospital weekly to check a fasting blood glucose level. Whilst there was no formally trained nutritionist or diabetes educator at the hospital, all women with GDM were given advice about nutrition from a doctor. Women with persistently raised fasting glucose (>7.0 mmol/l [126 mg/dl]) or 1-h post-prandial glucose (>11.1 mmol/l [200 mg/dl]) were commenced on insulin. At the time of the study, metformin was not licensed for use in pregnancy in Viet Nam and therefore was not used by any study participants.

The primary outcome for the study was increased neonatal growth, defined as large for gestational age (LGA),i.e., birth weight greater than the 90th population percentile for gestation and foetal gender. Birth weight was determined immediately after birth using digital scales accurate to the nearest 10 g (Tanita BD-590), and local birth-weight-for-gestation charts used. Other neonatal outcomes were as follows: preterm birth (<37 wk), death after study recruitment, small for gestational age (SGA), birth weight less than or equal to the tenth population percentile, intensive neonatal care, jaundice requiring phototherapy (initiated at bilirubin levels of >257 µmol/l at 25–48 h of age, >308 µmol/l at 49–72 h of age, or >342 µmol/l after 72 h of age), and symptomatic clinical neonatal hypoglycaemia, defined if there was a notion of hypoglycaemia in the medical record and either treatment with a glucose infusion or a recording of blood glucose level <2.6 mmol/l (46 mg/dl) within the first 48 h of life.

Maternal outcomes were induction of labour, primary caesarean section, postpartum haemorrhage (using the World Health Organization definition of >500 ml of blood loss in the first 24 h after birth [15]), severe perineal trauma (defined as laceration involving the anal sphincter), and preeclampsia (defined as blood pressure >140/90 mm Hg on at least two occasions and proteinuria >300 g in 24 h [16]).

### Sample Size

Sample size was based on estimation of LGA in the borderline group being 1.75 times higher that in the non-GDM group, as per the findings of the HAPO cohort study [4], upon which the IADPSG criterion was based [6]. The prevalence of GDM and LGA in this population had not been previously studied. Given prevalence in similar Asian populations, it was estimated that 7% of women would have GDM by the ADA criterion, and a further 7% would be borderline. If LGA prevalence were 10% in the normal group and 17.5% in the borderline group, for a power of 80% and two-sided significance of 0.05, approximately 2,295 women would be required in the cohort (using Fleiss with continuity correction). Allowing for 10% loss to follow-up, the study aimed to recruit at least 2,525 women.

### Statistical Analysis

All data were entered, verified, and hosted on a password-protected database. Any discrepancies and outlying results were reviewed. Identifying data for mothers and babies were removed. Data were analysed using STATA version 10.0 (StataCorp). All available data were used for analysis.

Categorical analysis compared mothers and babies in the three GDM strata (GDM, borderline, and normal). Maternal glycaemia was also analysed as a continuous variable, to determine the ability of the fasting, 1-h, and 2-h tests to predict adverse outcomes. This was felt to be an appropriate method for analysis, as categorisation of continuous variables risks losing information [17]. Adjusted odds ratios (aORs) for each increase in one standard deviation (SD) of glycaemia were calculated.

ANOVA univariate analysis was performed using a two-sample *t*-test with equal variances for continuous variables and a Pearson χ^2^ test to compare categorical variables. Multiple regression was performed with previously published confounding variables (purposeful selection). Unadjusted and adjusted odds ratios were calculated, with adjustment for age, body mass index (BMI) at the first antenatal visit, height at OGTT, partner's indoor smoking habit, family history of diabetes or hypertension, gestational age at OGTT, foetal sex, parity (not included in model for primary caesarean section), hospitalisation prior to delivery (not included in model for preeclampsia, primary caesarean section), and mean arterial blood pressure at the first antenatal visit (not included in model for preeclampsia).

Sensitivity analysis was conducted for women lost to follow-up.

## Results

### Sample Characteristics and Cohort Follow-Up

**Figure 1:**
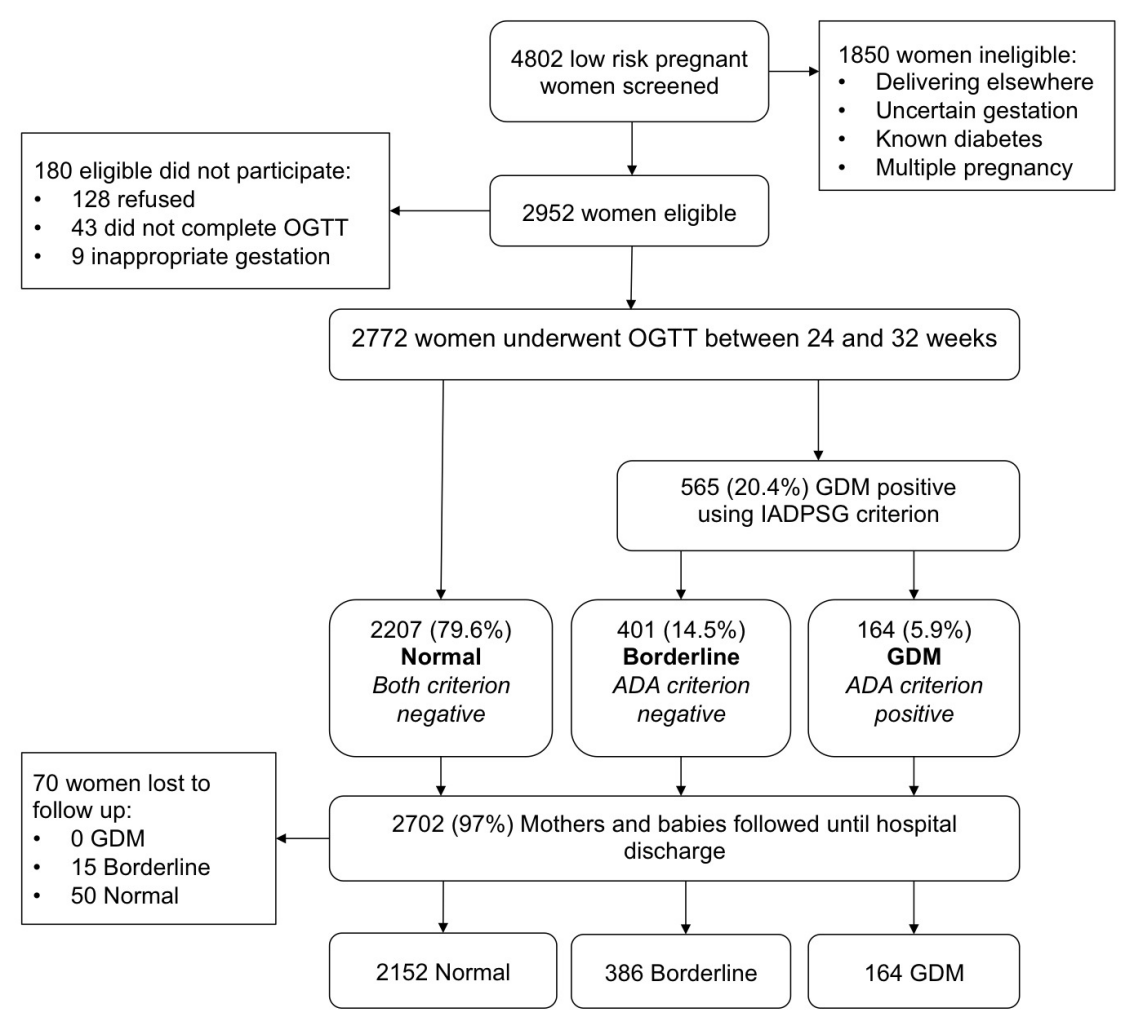
Flow chart of participants in cohort study.

The numbers of women approached and recruited into the study, as well as those lost to follow-up, are shown in Figure 1. From 1 December 2010 to 31 March 2011, 4,802 women presenting for routine antenatal care at Hung Vuong Hospital were screened for eligibility, with 2,952 women found eligible. The most common reason for ineligibility was planning to deliver elsewhere, with many city workers planning to return to their home province for delivery. Of eligible women, 2,824 consented to participate in the study, with 2,772 completing the OGTT (94% of eligible women). Complete birth and outcome data were available for 2,702 women and babies (97.5% of cohort). There were 70 women lost to follow-up, with no significant differences in basic demographic characteristics between them and the remainder of the cohort.

Maternal baseline characteristics are shown in Table 1. The mean gestation for the first antenatal visit was 12 wk (SD 6.6 wk) and mean gestation at OGTT was 28 wk (SD 1.7 wk). Women in the borderline GDM group were older, were of higher parity, had higher BMI, and were more likely to have a family history of diabetes women in the normal group. In addition to these risk factors, women with GDM were also more likely to have had GDM in a previous pregnancy, a prior caesarean section, a prior stillbirth or baby >4,000 g at birth, and/or a family history of hypertension. No women in the study stated that they smoked in pregnancy. Reported rates of pre-pregnancy hypertension were low in all groups (four women in the normal group, two women in the borderline group, and two in the GDM group), with no significant difference between groups.

**Table 1:** Maternal baseline characteristics.

Characteristic	Normal, *n* = 2,152 (Percent)	Borderline^a^, *n* = 386 (Percent)	*p*-Value	GDM, *n* = 164 (Percent)	*p*-Value
**Mean age, in years (SD)**	27.85 (4.73)	29.37 (4.89)	<0.001	31.21 (4.16)	<0.001
**Mean BMI** b **(SD)**	20.45 (2.63)	21.10 (2.99)	<0.001	21.81 (3.12)	<0.001
**Mean arterial pressure (SD)** c	77.65 (6.33)	78.45 (6.86)	0.02	78.46 (6.58)	0.12
**Gestation at OGTT, in weeks (SD)**	28.72 (1.75)	28.51 (1.69)	0.03	28.43 (1.79)	0.04
**Gestation at first antenatal visit, in weeks (SD)**	12.00 (6.53)	12.42 (6.69)	0.24	12.26 (7.01)	0.63
**Ethnicity Vietnamese**	2,046 (95.07)	370 (95.85)	0.51	158 (96.34)	0.47
**Education** d			0.85		0.54
Primary	162 (7.53)	31 (8.03)		14 (8.84)	
Secondary	1,392 (64.68)	244 (63.21)		99 (60.37)	
Tertiary	598 (27.79)	111 (28.76)		51 (31.10)	
**Parity = 0**	1,412 (65.61)	225 (58.29)	0.006	76 (43.34)	0.002
**Prior caesarean section**	169 (7.85)	34 (8.81)	0.52	28 (17.07)	<0.001
**Previous GDM**	6 (0.28)	1 (0.26)	0.95	3 (1.83)	0.002
**Previous stillbirth**	64 (2.97)	9 (2.33)	0.49	12 (7.32)	0.003
**Previous baby >4,000 g**	20 (0.93)	5 (1.30)	0.50	5 (3.05)	0.011
**Family history of diabetes** e	125 (5.81)	37 (9.59)	0.005	24 (14.63)	<0.001
**Family history of hypertension** e	372 (17.29)	70 (18.13)	0.69	48 (29.27)	<0.001

a IADPSG criterion for GDM positive, but ADA criterion negative.

b BMI (kg/m^2^) at first antenatal visit.

c Mean arterial pressure at first antenatal visit (mm Hg).

d Education level: primary, up to 5 y schooling; secondary, 6–12 y schooling; tertiary, >12 y schooling.

e Family history in first degree relatives.

### Maternal Glycaemia and Prevalence of Gestational Diabetes

Maternal glycaemia and prevalence of GDM are shown in Table 2. The median (range) of fasting, 1-h, and 2-h glucose levels was 4.4 (3.1–8.2), 7.9 (3.7–14.8), and 6.9 (3.3–15.2) mmol/l, respectively. There were 164 women (6.07%) with GDM by the ADA criterion and 550 women (20.36%) with GDM by the IADPSG criterion. Thus, 386 women were classified as borderline for the study purpose. There were 23 women (0.9%) with overt diabetes based on fasting glucose >7.0 mmol/l (126 mg/dl) or 2-h glucose >11.1 mmol/l (200 mg/dl). These women were included in the GDM group for analysis.

**Table 2:** Maternal glycaemia and prevalence of gestational diabetes.

Variable	Result (Median, in mmol/l) or *n*	Range (in mmol/l) or Percent
Fasting glycaemia	4.4	3.1–8.2
1-h glycaemia	7.9	3.7–14.8
2-h glycaemia	6.9	3.3–15.2
Normal (no GDM by either criterion)	2,152	79.64
GDM positive by IADPSG criterion	550	20.36
GDM positive by ADA criterion	164	6.07
Borderline group^a^	386	14.29

a Borderline group is women positive for GDM using the IADPSG criterion, but negative on the ADA criterion.

### Management of Gestational Diabetes

Of the 164 women diagnosed with GDM by the ADA criterion, 11 were commenced on insulin by the time of delivery (6.7%). Data were available on the method of glucose monitoring for 143 women (87% of the GDM group). 107 women performed home blood glucose monitoring. The frequency of self home monitoring varied, with 78 women testing glucose levels up to twice per week and three women testing more than four times per week. There were 36 women who underwent weekly or second weekly testing at the hospital or local clinic.

### Survival

There were no maternal deaths, 2,696 live births, six late-trimester stillbirths, and six neonatal deaths prior to hospital discharge in the cohort (Table 3). Three babies were born with major congenital malformations. There was no significant difference in perinatal mortality between the three groups; however, the study was not powered to demonstrate a difference in this outcome.

**Table 3:** Neonatal outcomes comparing referent group to borderline and GDM groups.

Outcome	Normal (Referent), *n* = 2,152 (Percent)	Borderline, *n* = 386 (Percent)	aOR^a^ (95% CI)	GDM, *n* = 164 (Percent)	aOR^a^ (95% CI)
Gestation at birth (weeks)^b^	38.85 (1.48)	38.64 (1.67)	0.20 (0.04–0.37)	38.31 (1.70)	0.53 (0.29–0.77)
Preterm delivery (<37 wk)	141 (6.55)	37 (9.59)	1.52 (1.03–2.24)	23 (14.02)	1.49 (1.16–1.91)
LGA^c^	253 (11.76)	62 (16.06)	1.31 (0.96–1.79)	31 (18.90)	1.16 (0.93–1.45)
SGA^d^	173 (8.04)	27 (6.99)	0.89 (0.58–1.37)	10 (6.10)	0.94 (0.67–1.32)
Clinical neonatal hypoglycaemia	15 (0.70)	9 (2.33)	3.34 (1.41–7.89)	23 (14.02)	4.94 (3.41–7.14)
Jaundice requiring phototherapy	65 (3.02)	16 (4.15)	1.39 (0.79–2.45)	7 (4.27)	1.16 (0.76–1.75)
Intensive neonatal care^e^	86 (4.0)	17 (4.40)	1.12 (0.65–1.91)	9 (5.49)	1.20 (0.83–1.73)
Perinatal death	9 (0.4)	3 (0.8)	1.68 (0.44–6.40)	0 (0)	NA

a Adjusted for age, BMI at OGTT, height at OGTT, indoor partner's smoking status, family history of diabetes, family history of hypertension, gestational age at OGTT, baby's sex, parity, hospitalisation prior to delivery, and mean arterial blood pressure at the first antenatal care visit.

b Mean (SD), mean difference with 95% confidence intervals.

c >90th population percentile for gestational age.

d <10th population percentile for gestational age.

e Intensive neonatal care defined as admission to the neonatal unit for care more intensive than normal newborn care and lasting more than 24 h, excluding suspected sepsis, observation, and feeding problems.

NA, not applicable.

### Neonatal Outcomes

Neonatal outcomes comparing women in the normal (referent) group to women in the borderline and GDM groups are shown in Table 3. Babies born to women in the GDM group were more likely to be born preterm (14.02% in the GDM group compared to 6.55% in the normal group, *p* = 0.002, aOR 1.49, 95% CI 1.16–1.91). Importantly, this relationship was also demonstrated in the borderline group, where 9.59% of babies were born preterm, which was also significantly increased from the rate seen in the normal group (*p* = 0.03, aOR 1.52, 95% CI 1.03–2.24). There was also a higher chance of developing clinical neonatal hypoglycaemia in the GDM group (14.02% in the GDM group compared to 0.70% in the normal group, *p*<0.001, aOR 4.94, 95% CI 3.41–7.14). Increased risk was also demonstrated in the borderline group, with 2.33% of babies requiring treatment for hypoglycaemia compared to 0.70% in the normal group (*p* = 0.01, aOR 3.34, 95% CI 1.41–7.89). The proportion of babies that were LGA was greater in the GDM (18.90%) and borderline (16.06%) groups compared to in the normal group (11.76%); however, this was not statistically significant once confounders, particularly BMI and age, were adjusted for: aOR 1.16 (95% CI 0.93–1.45) in the GDM group and aOR 1.31 (95% CI 0.96–1.79) in the borderline group. There was no significant difference in death, birth trauma, neonatal jaundice requiring treatment, or neonatal intensive care between the borderline or GDM group and the normal group.

### Maternal Outcomes

Maternal outcomes comparing women in the normal (referent) group to those in the borderline and GDM groups are shown in Table 4. Women with GDM were more likely to require antenatal hospitalisation (10.98% in women with GDM versus 4.88% in women without GDM, *p*<0.001, unadjusted odds ratio 2.25, 95% CI 1.40–3.61). Women in the GDM group were more likely to undergo primary caesarean section than women in the normal group (40.85% versus 33.46%, respectively); however, the increase was no longer significant after adjustment for confounders (aOR 1.13, 95% CI 0.94–1.37). Women in the borderline group had a slightly lower chance of primary caesarean section (31.35%, compared to 33.46% in the normal group), which also was not significant after adjustment (aOR 0.81, 95% CI 0.63–1.05). Women with GDM had an increased risk of induction of labour compared to women in the normal group; however, there was no significant difference for the borderline group compared to the normal group. The rate of preeclampsia overall was 1.70%, with only one case recorded in the GDM group and 39 in the borderline group, which was not significantly different to the normal women.

**Table 4:** Maternal outcomes comparing normal (referent) group to borderline and GDM groups.

Outcome	Normal, *n* = 2,152 (Percent)	Borderline, *n* = 386 (Percent)	aOR^a^ (95% CI)	GDM, *n* = 164 (Percent)	aOR^a^ (95% CI)
Preeclampsia	35 (1.63)	10 (2.59)	1.40 (0.68–2.89)	1 (0.61)	0.50 (0.18–1.39)
Primary caesarean section	720 (33.46)	121 (31.35)	0.81 (0.63–1.05)	67 (40.85)	1.13 (0.94–1.37)
Induction of labour	58 (2.84)	14 (3.88)	1.28 (0.69–2.34)	12 (7.64)	1.51 (1.08–2.11)
Severe perineal trauma^b^	52 (2.81)	10 (3.06)	1.11 (0.55–2.23)	4 (2.78)	1.0 (0.59–1.72)
Postpartum haemorrhage (>500 ml)	93 (4.32)	16 (4.15)	1.0 (0.60–1.69)	6 (3.66)	0.90 (0.58–1.38)

a Adjusted for age, BMI at OGTT, height at OGTT, indoor partner's smoking status, family history of diabetes, family history of hypertension, gestational age at OGTT, baby's sex, parity (not included in model for primary caesarean section), hospitalisation prior to delivery (not included in model for preeclampsia), mean arterial blood pressure at the first antenatal care visit (not included in model for preeclampsia).

b For women giving birth vaginally.

The results of the fasting, 1-h, and 2-h OGTT results were analysed as continuous variables for the major study outcomes. The odds ratio was calculated for each increase in one SD of glycaemia and is shown in Table 5. Significant relationships were demonstrated, as glycaemia across all three tests increased with the outcomes of LGA birth weight, neonatal hypoglycaemia, and preterm birth (<37 wk gestation). There was a significant decrease in the risk of SGA birth weight with rising 1-h and 2-h glycaemia, although this was not demonstrated with increasing fasting glycaemia. Labour induction also significantly increased with the 1-h and 2-h glycaemia results; however, again, this was not demonstrated for increases in fasting glycaemia. There was no statistically significant relationship between glycaemia and chance of primary caesarean section.

**Table 5:** Outcomes related to the 75-g oral glucose tolerance test results analysed as continuous variables.

Outcome	Plasma Glucose Level Odds Ratio^a^ (95% CI)		
	Fasting	1 h	2 h
Primary caesarean section	1.08 (0.99–1.17)	1.03 (0.94–1.12)	1.04 (0.96–1.14)
Preeclampsia	1.04 (0.87–1.25)	1.01 (0.83–1.23)	1.04 (0.87–1.24)
LGA	1.20 (1.08–1.34)	1.20 (1.07–1.33)	1.16 (1.04–1.30)
SGA	0.93 (0.80–1.09)	0.81 (0.70–0.95)	0.84 (0.72–0.97)
Labour induction	1.18 (0.96–1.44)	1.31 (1.05–1.62)	1.29 (1.07–1.57)
Neonatal hypoglycaemia	1.46 (1.18–1.81)	2.29 (1.74–2.99)	2.07 (1.66–2.58)
Neonatal jaundice requiring phototherapy	1.16 (0.95–1.40)	1.22 (0.98–1.51)	1.14 (0.93–1.40)
Preterm birth (<37 wk)	1.27 (1.11–1.45)	1.22 (1.06–1.42)	1.20 (1.04–1.38)
Intensive neonatal care	0.98 (0.81–1.20)	1.30 (1.07–1.57)	1.05 (0.87–1.26)

a Odds ratios were for an increase in the glucose level of one SD (0.42 mmol/l [7.6 mg/dl] for the fasting plasma glucose level, 1.6 mmol/l [28.9 mg/dl] for the 1-hr plasma glucose level, and 1.4 mmol/l [24.8 mg/dl] for the 2-hr plasma glucose level).

## Discussion

This study has demonstrated that there was an increase in neonatal morbidity amongst the extra cases of GDM diagnosed using the IADPSG criterion (the borderline group in this study). Major adverse findings demonstrated in the borderline group were an increase in the risk of preterm birth (<37 wk, aOR 1.52, 95% CI 1.03–2.24) and an increase in neonatal hypoglycaemia (aOR 3.34, 95% CI 1.41–7.89). These conditions are both potentially very serious in newborns, particularly in low-resource settings. We did not demonstrate any significant difference in maternal outcomes between the borderline and normal groups. The only significant maternal outcome difference between the GDM group and the normal group was an increase in the rate of induction of labour (aOR 1.51, 95% CI 1.08–2.11), which was frequently performed in women with GDM. We were not able to demonstrate an increase in the chance of primary caesarean section between the borderline group and the normal group (aOR 0.81, 95% CI 0.63–1.05), as was predicted from the data from the HAPO cohort [4]. It is of note, however, that primary caesarean section rates were much higher in the normal group (33.46%) in our study compared to that reported in the HAPO cohort (16.0%) [4]. Reasons for this high caesarean section rate are beyond the scope of this study, although similar high levels have been noted in other Asian metropolitan facilities [18], and there is evidence of a preference for caesarean section by both women and medical staff in some settings.

Introduction of universal screening for gestational diabetes in Viet Nam using the IADPSG criterion would identify over 20% of women as having GDM, compared to 6.1% if the ADA 2010 criterion were adopted.

A limitation of observational studies is the generalisability of these findings to similar populations. Whilst there was a high rate of participation and follow-up of eligible women, it is acknowledged that many women seeking care in this institution were not eligible. Women with high-risk pregnancies or known diabetes (including gestational) were excluded, and thus the prevalence of GDM in this study is likely to be an underestimate for the hospital, yet likely more reflective of the local metropolitan population. The study lacked blinding in outcome measurement as additional information on GDM management was obtained. Given the strong evidence to support identification and treatment of severe hyperglycaemia in pregnancy [8], it was felt unethical to deny treatment to women with more severe glucose intolerance. An attempt was made to minimise this as a source of bias by having a structured questionnaire for outcome measurement, and the health providers completing patient records were unaware of the study outcomes. The study is strengthened by the prospective design, defined aims, high rate of follow-up for women and babies in the cohort (97.5% of women who underwent OGTT screening), and ability to adjust for several possible confounding variables. The use of local birth weight for gestation percentile charts could be criticised as not representing optimal growth. Until the release of the proposed World Health Organization birth weight charts for gestational age, it was felt the local charts were appropriate. The mean birth weight for Vietnamese females born at 40 wk is 3,166 g and for males is 3,330 g, the 90th percentiles being 3,650 g and 3,760 g, respectively. These figures are significantly lower than the mean Australian population birth weight standards at 40 wk, 3,450 g for females and 3,600 g for males, and the 90th percentiles 4,000 g and 4,170 g, respectively [19]. Babies born to mothers with GDM with normal birth weight have been found to have higher body fat percentage than those born to mothers with normal glucose tolerance [20], and it may be that this is a better measure of metabolic disturbance in the neonate than weight for gestational age or weight greater than 4,000 g. We lacked the resources to measure body fat composition, and so LGA has been used as a proxy measure for increased neonatal growth.

The number of women who commenced on insulin, 11 (6.5% of the GDM group), is lower than reported from other settings. Whilst diagnostic criteria and thresholds for commencement of insulin differ across settings, rates of reported insulin usage in women with GDM range from 17% to 40% [3],[21],[22]. The lower rates of insulin use in this study are likely related to the less frequent monitoring, with 80% of women monitoring glucose levels twice a week or less often. This may have contributed to the relatively high rate of neonatal hypoglycaemia seen in the GDM group (14.02%), although it is of note that of the 141 babies born preterm to mothers in the normal group, only 15 developed clinical neonatal hypoglycaemia.

Inadequate or less-intensive treatments for GDM in retrospective studies from India [23] and South Africa [24] have been found to be associated with increased perinatal mortality compared to treatment deemed adequate. Neither of these studies controlled for confounding, and both studies included women with minimal antenatal care diagnosed with GDM very late in gestation. We did not demonstrate a difference in perinatal mortality in this study between the women with and without GDM, although this study was underpowered to demonstrate a difference in this outcome. In this study all women had regular and frequent antenatal care from an average of 12 wk gestation, and education and literacy rates were relatively high, with over 90% of women having completed at least primary school. Low education and infrequent antenatal care are known to be associated with stillbirth in this population [25], thus the findings from this study may not be generalisable to the Vietnamese population as a whole.

Our study has confirmed that GDM occurs at much lower BMI in Vietnamese women than in Caucasian women. The mean BMI for women in the GDM group was 21.0 kg/m^2^ and in the borderline group was 20.22 kg/m^2^. Using the World Health Organization Asian reference categories [26], 15.5% of women were classified as overweight (BMI 23–27.5 kg/m^2^) and 1.8% of women obese (BMI>27.5 kg/m^2^). An Australian study examining differences in insulin resistance between ethnic groups in women with GDM found a much lower BMI in Asian women than in other ethnic groups for the same degree of insulin resistance [27]. It has also been shown that as BMI increases in Asian women, there is a significantly greater increase in insulin resistance than in Caucasian women [28].

The low rates of overweight and obesity in both the GDM and normal groups in our study resulted in only a 4.7% population attributable fraction of overweight and obesity for GDM in Vietnamese women. In North America it has been estimated that the population attributable fraction of overweight and obesity for GDM is 46.2% [29]. This raises the question of why the rates of GDM are so high in this population. Whilst GDM is likely multifactorial in origin, it has been proposed that genetic predisposition [30] and/or foetal programming [31] are likely to be of key importance.

In the HAPO cohort study the mean BMI was 27.7±5.1 kg/m^2^, with means from study centres ranging from 24.4 to 29.9 kg/m^2^ [4]. We feel our study is important as it demonstrates that in women with lower BMI, the frequency of adverse outcomes secondary to hyperglycaemia is lower than in populations where the mean BMI is in the overweight range.

In Western populations there appears to be a synergistic effect of GDM and obesity. In a review of 3,789 women with GDM from the United States, the risk of composite adverse neonatal outcome (birth weight >4,000 g, birth trauma, shoulder dystocia, hypoglycaemia, or jaundice) increased from 20.4% for normal weight (BMI 18.5–24.9 kg/m^2^) to 35.9% in women with morbid obesity (*p*<0.001) [32]. Given the low rate of overweight and obesity, our study was underpowered to replicate these findings.

In this study the rate of preeclampsia was low, 1.70%, with no difference between the GDM groups. Hyperglycaemia in pregnancy has been associated with the development of preeclampsia [9], with the ACHOIS trial demonstrating that treatment of mild hyperglycaemia can decrease preeclampsia (relative risk 0.70, 95% CI 0.51–0.95) [3]. Whilst this association was also demonstrated in the HAPO cohort [4], post hoc analysis demonstrated the relationship between preeclampsia and BMI to be stronger than that with hyperglycaemia [33].

Despite the fact that Asian expatriate women have higher rates of GDM than Caucasian women [34],[35], they appear to have less preeclampsia. In a retrospective review of 902,000 women giving birth in New York City from 1995 to 2003, East Asian women had the lowest rates of preeclampsia (1.4% compared to 3.2% overall) [36]. The low prevalence of preeclampsia and the low BMI in our study may have resulted in type 1 error in failing to demonstrate an association with GDM.

The proposed IADPSG guidelines have generated much controversy and discussion amongst clinicians globally working in the area of GDM. Our study demonstrates a 300% increase in GDM prevalence if the IADPSG criterion is adopted in Viet Nam. Retrospective studies from several countries also demonstrate that use of the IADPSG criterion will significantly increase workload. In Australia, prevalence of GDM would increase from 7.7% to 9.4% [37], in Japan, from 2.4% to 6.6% [38], and in the United Arab Emirates, from 12.9% to 37.7% [39].

### Conclusion

The proposed IADPSG criterion for GDM would identify more women as having GDM who are at risk of having a preterm birth or a baby requiring treatment for neonatal hypoglycaemia. Before recommending that this screening method be adopted in Viet Nam, these findings need to be balanced against the ability of a low-resource hospital to manage such a large number of women with GDM. Without evidence of the benefit of treatment for the women in the borderline group, it is difficult to recommend adoption of the IADPSG guidelines in Viet Nam at present. The current low prevalence of overweight and obesity in pregnant women may be offering some protection against other adverse outcomes associated with milder degrees of GDM. The long-term significance of a diagnosis of GDM using the IADPSG definition in predicting future risk of type 2 diabetes mellitus is not known. Future research needs to be directed at examining the short- and long-term benefits as well as potential harms and opportunity costs of screening and treating GDM in low-income settings before “universal” screening in pregnancy can be recommended.

## Abbreviations

ADA: American Diabetes Association
aOR: adjusted odds ratio
BMI: body mass index
GDM: gestational diabetes mellitus
HAPO: Hyperglycaemia and Adverse Pregnancy Outcomes
IADPSG: International Association of the Diabetes and Pregnancy Study Groups
LGA: large for gestational age
OGTT: oral glucose tolerance test
SD: standard deviation
SGA: small for gestational age
